# Another Antidote to Arsenic

**Published:** 1880-08

**Authors:** 


Another Antidote to Arsenic.—Dr. McCaw, a Canadian physician, suggests the following formula as one not generally known for an antidote to arsenic, and claims for it preference over all others for two reasons, namely, because it forms the surest antidote, and because the ingredients are always accessible :

R.—Tincture of chloride of iron............℥i
Bicarbonate of soda or potash .....℥i
Tepid water..................a teacupful.

These are mixed. The sesquioxide of iron is immediately formed in a solution of chloride of sodium.

The mixture may be given almost *ad libitum.—Southern Medical Record.*

